# Correlation between dental caries experience and the level of *Streptococcus mutans* and lactobacilli in saliva and carious teeth in a Yemeni adult population

**DOI:** 10.1186/s13104-020-04960-3

**Published:** 2020-02-27

**Authors:** Sabah A. Sounah, Ahmed A. Madfa

**Affiliations:** 1 Department of Restorative and Prosthodontics, College of Dentistry, University of Sciences and Technology, Sanaa, Yemen; 2 Department of Conservative Dentistry, Faculty of Dentistry, Thamar University, Dhamar, Yemen; 3 Department of Restorative Dental Science, College of Dentistry, University of Hail, Hail, Kingdom of Saudi Arabia

**Keywords:** *Streptococcus mutans*, Lactobacilli, Quantitative, real-time PCR, Caries risk, Epidemiology, Yemeni population

## Abstract

**Objective:**

This study aimed to determine the relative amounts of *Streptococcus mutans* (*SM*) and lactobacilli (LBs) and their relationship with dental caries among a Yemeni adult population.

**Results:**

A positive correlation appeared between *SM* and LB counts from saliva and caries tissue samples and the decayed, missing and filled teeth (DMFT) score (*p* < 0.05). There was a significant correlation between *SM* and LBs in caries tissue (*p* < 0.05). However, there was no significant difference between *SM* and LBs isolated from saliva samples (*p* > 0.05). The number of *SM* and LBs in subjects with active caries was significantly higher than that in those without active caries (*p* < 0.05). There was no significant difference between the daily habits and *SM* and LB or DMFT scores (*p* > 0.05), except for a significant difference between brushing frequency and DMFT score (*p* < 0.05).

## Introduction

Dental caries is a major infectious disease affecting the majority of the world’s population [1]. It is considered one of the most prevalent diseases, particularly in socially underdeveloped communities [2].

In the past few decades, extensive research has provided important information about the link between dental caries and salivary bacteria [3], and some studies have revealed a significant association between salivary levels of *Streptococcus mutans* (*SM*) and subsequent onset of caries [4]. Existing data on the possible association between lactobacillus (LB) saliva levels and the onset of caries are less convincing [5]. Therefore, salivary LB levels might be indirectly related to caries progression [6].

Because oral bacteria are considered one of the aetiologic factors involved in caries development [7], various microbial studies have been conducted to better understand this dental problem. However, there is a lack of available data on the detection of dental caries bacterial diversity in the Yemeni adult population. In addition, culture-independent studies describing the number of distinct microorganisms in the saliva and carious lesions of Yemeni adults have not yet been published. Thus, information about the correlation between the distribution of *SM* and LBs and caries development is needed. The current study was planned to investigate the adult population of Yemen to determine the relative amounts of *SM* and LBs and to study their correlation with dental caries using quantitative real-time PCR.

## Main text

### Methods

#### Study design and population

Ethics committee of the Faculty of Medicine and Health Sciences at UST approved this study (MECA No. 2017/01). The study sample comprised males and females with an average age of 24 years referred for treatment to the dental polyclinics of the Dental College at UST. The inclusion criteria comprised subjects with good general health, more than three carious teeth for caries-active (CA) subjects and sound teeth or one initial non-cavitation for caries-free (CF) individuals. The exclusion criteria were subjects who had any of the following conditions: systemic disorders or use of medication that might influence the dentition; mental disability; a history of antibiotic or fluoride treatment; consultation for orthodontic reasons or under orthodontic treatment. The final sample size in this study consisted of 40 individuals (20 CF and 20 CA). Written informed consent was obtained from all individual participants included in the study.

#### Clinical examination and daily habits interview

All examinations were performed using dental mirrors and a dental explorer. According to the WHO dental caries diagnostic criteria, clinical oral health status was assessed, and decay (D), loss due to dental caries (M) and filled (F) teeth (T) (DMFT) were observed. One examiner conducted the clinical examination using the DMFT standard. A bite-wing radiograph was obtained for each subject to confirm the depth of the lesion [8].

After the clinical examination, all subjects received interviews about their daily oral health habits. This study used a questionnaire survey adapted from earlier studies [9, 10]. Subjects were asked about the practice of oral hygiene and the frequency of sugar consumption.

#### Sample collection and extraction of genomic DNA

Twenty specimens were excavated from carious teeth of CA individuals using a sterilized, sharp dental spoon excavator. The lesion in each tooth was excavated, suspended in alcohol, and kept at − 20 °C until use. One gram of the carious dentine fragment was weighed and initially dispersed manually by vortexing for 20 s prior to extraction of bacterial DNA [11, 12].

Forty saliva specimens were collected for this study, 20 specimens from CF individuals and 20 specimens from CA subjects. The saliva collection was scheduled for early morning at the beginning of the day before the patients had breakfast. Each patient was asked to swallow pre-existing saliva and then chew a standard piece of paraffin wax, and 5 mL of stimulated saliva was collected into a 50-mL sterile tube, which was then taken directly to the laboratory and kept at − 20 °C until it was used [2, 8]. Total bacterial genomic DNA was then extracted from each saliva sample using a modification based on the instructions of a published study [2, 8, 13] (Additional file 1).

#### Bacterial strains

The number of oral bacterial strains from two internal control species was observed in this study to construct a quantitative standard curve. *SM* and LBs were isolated, identified, and confirmed by conventional PCR test [2]. Bacterial genomic DNA was extracted and purified according to the Qiagen bacterial extraction protocol and the company’s extraction guide (Qiagen, Hilden, Germany).

#### Quantitative real-time PCR assays

For quantification of the total level of bacterial samples, qPCR for the targeted 16S rRNA gene was obtained on an ABI 7000 real-time PCR instrument (Applied Biosystems, California, United States) in a total reaction volume of 20 µL. The specific forward and reverse primers used in this study were as described and validated elsewhere [13, 14] (Additional file 2). The real-time PCR assay was performed in a total volume of 20 µL consisting of 10 µL of 2 × Eva Green qPCR Mix Plus ROX (Solis Bio Dyne, Estonia), 2 µM each forward and reverse primer, 5 µL of template DNA and 3 µL of DNA/RNAse-free water. Each sample was tested twice. Conditions of thermal cycling for all qPCR assays were set as follows: one cycle at 95 °C for 15 min, followed by 40 cycles of denaturation for 15 s at 95 °C, primer annealing for 20 s at 60 °C for *SM* and LBs, and elongation for 20 s at 72 °C for *SM* and LBs. For the construction of quantification standard curves, the qPCR reaction mixture contained a total volume of 20 µL, which consisted of 10 × Eva Green qPCR Mix Plus ROX (Solis Bio Dyne, Estonia), 2 µM each forward and reverse primer, 5 µL of template genomic DNA from the aforementioned bacterial strains and 3 µL DNA/RNAse-free water (concentration ranged from 10 ng/µL to 10 fg/mL) [14]. The results are expressed as the number of cells per mL, according to the calculation method described elsewhere [13].

#### Statistical analysis

All data were collected and analysed statistically by Statistical Package for Social Science version 23.0 (SPSS Inc., Chicago, IL). Due to unmet assumptions of normal distribution after results of the Shapiro–Wilk test (*p* < 0.05), nonparametric tests were used. Correlations between both bacteria and the DMFT index were analysed by the nonparametric Spearman correlation coefficient. Kruskal–Wallis and Mann–Whitney U tests were used to determine statistically significant differences between the groups. A *p* value less than 0.05 was considered significant.

### Results

**Fig. 1:**
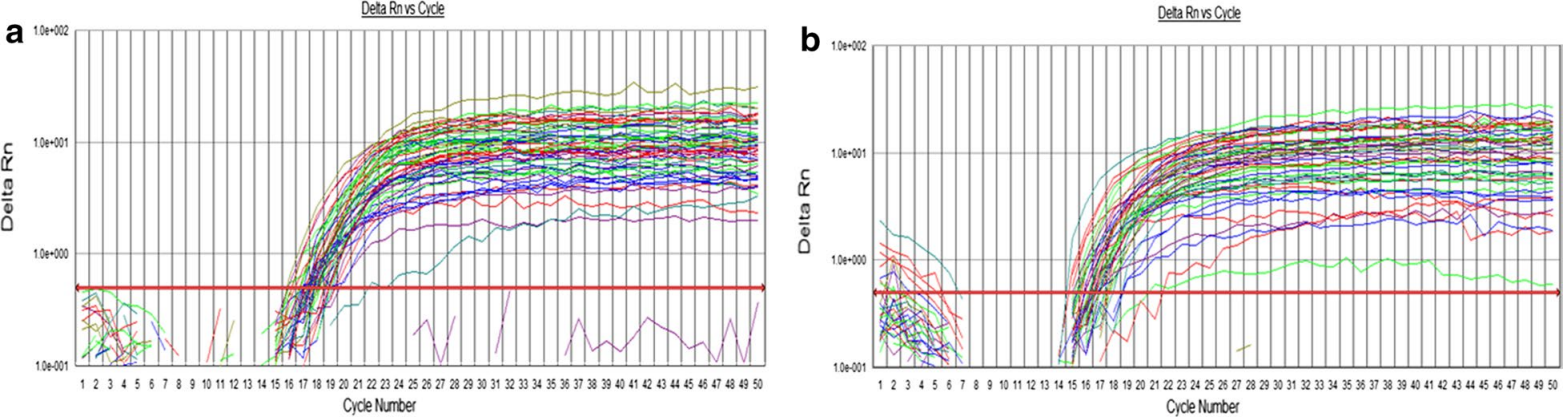
Quantification graph of *SM* (**a**) and LB (**b**) from saliva and caries tissue samples using 16S RNA specific primers to quantification of specific Bacteria in caries active and caries free. Successful amplification was achieved in all samples whilst the NTC showed negative amplification with no detectable Ct value

Based on qPCR results, *SM* and LBs were detected in 100% of the Yemeni adults (Fig. 1; Additional files 3 and 4). Table 1 displays the correlations of the relative amounts of *SM* or LBs in both saliva and caries lesions, with DMFT scores. A positive correlation was found between *SM* isolated from CA saliva and caries tissue samples and the DMFT score (*p* < 0.05), whereas no significant relationship was found between *SM* isolated from caries tissue and the DMFT score (p > 0.05). No significant relationship was found between LBs isolated from saliva and caries tissue samples with the DMFT score (*p* > 0.05). However, there was a significant inverse correlation between *SM* and LBs isolated from caries tissue (*p* < 0.05).

**Table 1:** Correlation coefficients of bacteria and caries development in caries-active individuals

	SM	LB
Saliva samples		
SM	1.00	0.324
LBs	0.324	1.00
DMFT	0.021*	0.703
Caries tissue samples		
SM	1.00	0.174
LBs	0.056*	1.00
DMFT	0.174	0.833

* *P* < *0.05*

When comparing both groups, the number of *SM* and LBs in subjects with active caries was significantly higher than that in those without active caries (*p* < 0.05), as presented in Table 2. Furthermore, significant differences appeared between samples of saliva from CF subjects and both saliva and caries lesion samples from CA subjects (*p* < 0.05). However, there was no difference between the number of bacteria isolated from saliva and caries lesion samples from CA subjects (*p* > 0.05). Significantly higher DMFT scores were found in CA subjects than in CF individuals (*p* < 0.05). Decayed, missing, and filled teeth in relation to the numbers of *SM* and LBs and the DMFT scores also showed significant differences (*p* < 0.05), as displayed in Table 2.

**Table 2:** Comparison of the relative amounts of tested bacteria with DMFT index in the caries-active and caries-free groups by using Kruskal–Wallis and Mann–Whitney U tests

	Caries-active	Caries-free	*p*	CF saliva	CA saliva	Caries tissue	*p*
	Median			Median			
*SM*	4.1 × 10^6^	4.9 × 10^6^	0.001*^a^	4.4	4.6 × 10^6^	4.4 × 10^6^	0.006*^b^
LBs	3.3 × 10^6^	3.5 × 10^6^	0.000*^a^	3.3	3.2 × 10^6^	3.3 × 10^6^	0.001*^b^
DMFT	8.0	0	0.000*^a^	0	9.0		0.000*^b^
CF saliva				–	0.000*^c^	0.000*^c^	
CA saliva				0.016^*d^	–	1.000	
Carious tissue				0.004^*e^	1.000	–	

	*SM*	LB	DMFT				
Decayed	0.001^*f^	0.000^*f^	0.000^*g^				
Missing	0.620	0.275	0.016^*g^				
Filled	0.174	0.194	0.032^*g^				

* p < 0.05, ^a^SM, LBs, and DMFT score in caries-active and caries-free subjects; ^b^SM, LBs, and DMFT score in the three types of samples (caries-free saliva, caries-active saliva, and carious tissue); ^c^difference between samples in relation to DMFT score; ^d^difference between samples in relation to SM and ^e^to LBs, ^f, g^decayed, missing, and filled in relation to SM, LBs, and DMFT score

There were no significant differences between the daily habits (chewing khat, brushing, flossing, frequency of eating sweets, and drinking soft drinks) and *SM* and LB levels and DMFT scores (*p* > 0.05), except for brushing frequency and DMFT scores, which showed a significant difference between the two groups (*p* < 0.05) (Additional file 5).

### Discussion

In the present study, saliva was particularly selected as the sampling medium to assess the microbial aspect of dental caries. Salivary *SM* and LBs enumeration may be considered a reflection of an individual’s oral load of these bacteria [15–17]. It is expected that the use of saliva samples in this study provides a generalized picture of the microbial load of these species, and when measuring relative counts, the risk trend of caries in all dentitions can be emphasized. In addition, sampling from caries lesions for CA individuals was considered. Other studies also reported the detection of elevated numbers of LBs and S*M* in carious samples by using qPCR [18].

In this study, the detection of *SM* and LBs in the salivary and carious tissue samples was 100%, which is consistent with the results of Kishi et al. [19], who revealed a frequent detection of *SM* in saliva samples (100%), which was significantly related to the DMFT score.

Our results also indicated that the detection of *SM* and LBs was in the CA and CF saliva; however, the levels of both kinds of bacteria appeared to be higher in the CA subjects than in the CF subjects. Individuals with lower concentrations showed a significantly lower mean number of decayed surfaces than did the individuals with higher concentrations of *SM* in their saliva [20]. The current study also showed an inverse correlation between the levels of *SM* and LBs, but the difference was not statistically significant. These findings are in agreement with Chen Lin et al. [14], who found no significant association between *SM* and LBs (Additional files 1, 2, 3, 4, 5).

Positive associations were noted between caries development and the level of *SM* in saliva samples. These results agree with the studies conducted by other authors who reported a positive association between the concentration of *SM* in saliva and dental caries [19, 21, 22]. A negative correlation between *SM* and LBs in caries tissue samples was supported by the hypothesis that *SM* and LBs are more competitive under certain acidic conditions [23]. A result of an in vitro study, which was supported by this study, showed that LBs were capable of preventing the growth of *SM* [24].

LBs are prominent organisms in dental caries, and a relatively high proportion is found in cavity lesions, suggesting that their role in caries may lie in progression and advanced caries rather than in initiation of disease [25]. Simón-Soro et al. [26] took samples from carious enamel and carious dentin and revealed that the proportion of *SM* was greater in dentin caries, whereas LB abundance was greater in deep dentin lesions. This result was in agreement with our findings, in which the level of LBs appeared to be higher in carious tissue than in saliva among the CA group. However, another study carried out on adults revealed a strong relationship between root caries and the level of salivary LBs [27].

The current study found that the bacterial counts and DMFT index were not influenced by daily oral habits with the exception for tooth brushing, for which there was a positive relationship with DMFT score. These findings agreed with a previous study [28].

It can be concluded that *SM* and LBs could be detected quantitatively and sensitively by qPCR. Higher *SM* and LB counts were observed in CA subjects, and the lowest counts were observed in the CF subjects. *SM* and LB counts showed a positive correlation with DMFT scores in the group with dental caries, which likely reinforces their relationship with dental caries development.

## Limitations

A cross-sectional study design was used in the present study to determine the correlations among *SM*, LBs, and caries rates. However, a single saliva sample records the microbial counts at one particular point of time, and it is well understood that dental caries develops over a considerable period of time, during which bacterial counts could fluctuate in response to the changing oral environment [22]. Moreover, the number of CF and CA samples studied was limited; further study with a larger sample should be performed to confirm these results.

## Supplementary information

**Additional file 1: Figure S1.** Distribution of the study sample.

**Additional file 2: Table S1.** Bacterial-specific primers for quantitative real-time polymerase chain reaction.

**Additional file 3: Table S2.** Mean (standard deviation [SD]) of *Streptococcus mutans*, lactobacilli, and DMFT score in caries-free and caries-active subjects.

**Additional file 4: Figure S2** PCR successful amplification of positive control for *SM* and LB species with 256 bp and 430 pb, respectively. Electrophoretic separation of a fragment of 415 bp of gene gtfB and 223 16S rDNA in 2% agarose gel amplified by means of PCR. Lane 1 corresponds to a standard molecular size of 1 kb. Lanes 2 and 3 correspond to amplification, using the DNA of *S. mutans* UA159. Lanes 4 and 5 correspond to amplifications using DNA.

**Additional file 5: Table S3.** Median (25th, 75th percentiles) *Streptococcus mutans*, lactobacilli, and DMFT index in relation to different daily habits in caries-free and caries-active subjects.

## Data Availability

The data used and/or analysed during the present study is included as supplementary file. Any additional data and material are available from the corresponding author on reasonable request.

## Abbreviations

SM: *Streptococcus mutans*
LBs: Lactobacilli
LB: *Lactobacillus*
qPCR: Quantitative polymerise chine reaction
CA: Caries-active
CF: Caries-free
DMFT: Decayed, missing and filled teeth
