# Supplementary figures and images for: Correlation between dental caries experience and the level of Streptococcus mutans and lactobacilli in saliva and carious teeth in a Yemeni adult population
Source: BMC Res Notes. 2020 Feb 27;13:112. doi: 10.1186/s13104-020-04960-3 (PMC7045487; doi:10.1186/s13104-020-04960-3)

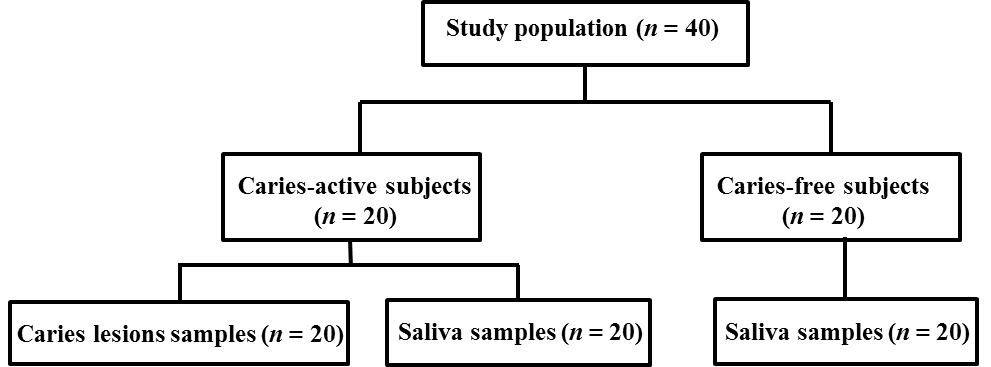


**Additional Figure S1** Distribution of the study sample

Supplement: Supplementary file 1 — Additional file 1: Figure S1. Distribution of the study sample. [file 13104_2020_4960_MOESM1_ESM.docx]
