# Supplementary material for: Correlation between dental caries experience and the level of Streptococcus mutans and lactobacilli in saliva and carious teeth in a Yemeni adult population
Source: BMC Res Notes. 2020 Feb 27;13:112. doi: 10.1186/s13104-020-04960-3 (PMC7045487; doi:10.1186/s13104-020-04960-3)
Supplement: Supplementary file 2 — Additional file 2: Table S1. Bacterial-specific primers for quantitative real-time polymerase chain reaction. [file 13104_2020_4960_MOESM2_ESM.docx]

**Additional Table S1** Bacterial-specific primers for quantitative real-time polymerase chain reaction

| Targeted bacteria | Primers (50e30) | Amplicon sizes (bp) | Targets |
| --- | --- | --- | --- |
| SmF5 SmR4 | AGCCATGCGCAATCAACAGGTT  CGCAACGCGAACATCTTGATCAG | 415 | gtfB |
| *Lactobacillus* spp. | TGGAAACAGATGCTAATACCG  CGTCCATTGTGGTAGATTCCCT | 223 | 16S rDNA |
