# Supplementary material for: Correlation between dental caries experience and the level of Streptococcus mutans and lactobacilli in saliva and carious teeth in a Yemeni adult population
Source: BMC Res Notes. 2020 Feb 27;13:112. doi: 10.1186/s13104-020-04960-3 (PMC7045487; doi:10.1186/s13104-020-04960-3)
Supplement: Supplementary file 3 — Additional file 3: Table S2. Mean (standard deviation [SD]) of Streptococcus mutans, lactobacilli, and DMFT score in caries-free and caries-active subjects. [file 13104_2020_4960_MOESM3_ESM.docx]

**Additional Table S2** Mean (standard deviation [SD]) of *Streptococcus mutans*, lactobacilli, and DMFT score in caries-free and caries-active subjects

| **Variables** | | **Mean ±SD** |
| --- | --- | --- |
| **CF subjects** | SM | 4.2×10^6^±2.3×10^5^ |
|  | LBs | 3.4×10^6^±4.3×10^5^ |
|  | DMFT | 0 |
| **CA subjects** | SM in saliva | 4.7×10^6^±3.9×10^5^ |
|  | LBs in saliva | 3.1×10^6^±2.8×10^5^ |
|  | SM in carious tissue | 4.8×10^6^±4.0×10^5^ |
|  | LBs in carious tissue | 3.6×10^6^±3.3×10^5^ |
|  | DMFT | 8.3 ± 2.0 |
