# Supplementary material for: Correlation between dental caries experience and the level of Streptococcus mutans and lactobacilli in saliva and carious teeth in a Yemeni adult population
Source: BMC Res Notes. 2020 Feb 27;13:112. doi: 10.1186/s13104-020-04960-3 (PMC7045487; doi:10.1186/s13104-020-04960-3)
Supplement: Supplementary file 4 — Additional file 4: Figure S2 PCR successful amplification of positive control for SM and LB species with 256 bp and 430 pb, respectively. Electrophoretic separation of a fragment of 415 bp of gene gtfB and 223 16S rDNA in 2% agarose gel amplified by means of PCR. Lane 1 corresponds to a standard molecular size of 1 kb. Lanes 2 and 3 correspond to amplification, using the DNA of S. mutans UA159. Lanes 4 and 5 correspond to amplifications using DNA. [file 13104_2020_4960_MOESM4_ESM.docx]

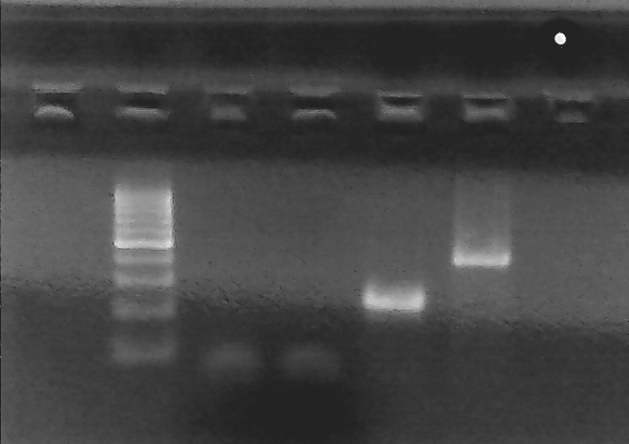


**Additional Figure S2** PCR successful amplification of positive control for *SM* and LB species with 256 bp and 430 pb, respectively. Electrophoretic separation of a fragment of 415 bp of gene gtfB and 223 16S rDNA in 2% agarose gel amplified by means of PCR. Lane 1 corresponds to a standard molecular size of 1 kb. Lanes 2 and 3 correspond to amplification, using the DNA of *S. mutans* UA159. Lanes 4 and 5 correspond to amplifications using DNA from saliva.
