# Supplementary material for: Correlation between dental caries experience and the level of Streptococcus mutans and lactobacilli in saliva and carious teeth in a Yemeni adult population
Source: BMC Res Notes. 2020 Feb 27;13:112. doi: 10.1186/s13104-020-04960-3 (PMC7045487; doi:10.1186/s13104-020-04960-3)
Supplement: Supplementary file 5 — Additional file 5: Table S3. Median (25th, 75th percentiles) Streptococcus mutans, lactobacilli, and DMFT index in relation to different daily habits in caries-free and caries-active subjects. [file 13104_2020_4960_MOESM5_ESM.docx]

**Additional Table S3** Median (25th, 75th percentiles) *Streptococcus mutans*, lactobacilli, and DMFT index in relation to different daily habits in caries-free and caries-active subjects

|  | Median | | | | | | *P* |
| --- | --- | --- | --- | --- | --- | --- | --- |
|  | **Chewing khat frequency** | | | | | |  |
|  | Non (n=25) | Daily (n=5) | | Weekly (n=2) | Monthly (n=8) |  |  |
| SM | 4.3×10^6^ (4.1×10^6^, 4.5×10^6^) | 4.8×10^6^ (4.1×10^6^, 4.5×10^6)^ | | 4.3×10^6^ (4.7×10^6^) | 4.5×10^6^ (4.6×10^6^, 4.1×10^6^) |  | .889 |
| LBs | 3.7×10^6^ (3.4×10^6^, 3.1×10^6^) | 3.5×10^6^ (3.4×10^6^, 4.7×10^6^) | | 3.2×10^6^ (3.2×10^6^) | 3.6×10^6^ (3.3×10^6^, 3.6×10^6^) |  | .481 |
| DMFT | .000 (.000, 9) | 7 (.000, 9.5) | | 9 (8) | 2.5 (.00, 8) |  | .459 |
|  | **Brushing frequency** | | | | | |  |
|  | More than once per day (n=16) | | Daily (n=10) | Weekly (n=6) | Non (n=8) |  |  |
| SM | 4.4×10^6^ (4.9×10^6^, 4.4×10^6^) | | 4.1×10^6^ (4.4×10^6^, 4.8×10^6^) | 4.1×10^6^ (4.8×10^6^, 4.6×10^6^) | 4.6×10^6^ (4.0×10^6^, 4.2×10^6^) |  | .488 |
| LBs | 3.8×10^6^ (3.8×10^6^, 4.6×10^6^) | | 3.4×10^6^ (3.8×10^6^, 3.2×10^6^) | 3.1×10^6^ (3.7×10^6^, 3.1×10^6^) | 3.5×10^6^ (3.3×10^6^, 3.6×10^6^) |  | .309 |
| DMFT | .000 (.000, 6) | | .000 (.000, 7.7) | 8.5 (7.2, 9.5) | 9 (7, 9.7) |  | .003* |
|  | **Flossing frequency** | | | | | |  |
|  | More than once per day (n=8) | | Daily (n=4) | Weekly (n=13) | Non (n=15) |  |  |
| SM | 4.1×10^6^ (4.8×10^6^, 4.2×10^6^) | | 4.0×10^6^ (4.6×10^6^, 4.2×10^6^) | 4.0×10^6^ (3.1×10^6^, 4.8×10^6^) | 4.6×10^6^ (4.4×10^6^, 4.3×10^6^) |  | .446 |
| LBs | 3.7×10^6^ (3.6×10^6^, 3.9×10^6^) | | 3.2×10^6^ (3.8×10^6^, 3.6×10^6^) | 3.4×10^6^ (3.4×10^6^, 3.1×10^6^) | 3.6×10^6^ (3.3×10^6^, 3.0×10^6^) |  | .716 |
| DMFT | 7.5 (1.7, 9) | | 4 (.000, 8) | .000 (.000, 6.5) | 7 (.000, 9) |  | .393 |
|  | **Sweets consumption frequency** | | | | | |  |
|  | More than once per day (n=12) | | Daily (n=22) | Weekly (n=2) | Monthly (n=1) | Non (n=3) |  |
| SM | 4.5×10^6^ (4.0×10^6^, 4.3×10^6^) | | 4.5×10^6^ (4.3×10^6^, 4.9×10^6^) | 4.4×10^6^ (4.1×10^6^) | 4.4×10^6^ (4.4×10^6^, 4.4×10^6^) | 4.8×10^6^ (4.9×10^6^) | .720 |
| LBs | 3.9×10^6^ (3.5×10^6^, 3.7×10^6^) | | 3.1×10^6^ (3.7×10^6^, 3.5×10^6^) | 3.3×10^6^ (3.4×10^6^) | 3.7×10^6^ (3.7×10^6^, 3.7×10^6^) | 3.6×10^6^ (3.6×10^6^) | .087 |
| DMFT | 6.5 (.000, 9.7) | | .000 (.000, 8.2) | 4.5 (.000) | .000 (.000, .000) | 7 (.000) | .802 |
|  | **Soft drink consumption frequency** | | | | | |  |
|  | More than once per day (n=8) | | Daily (n=18) | Weekly (n=4) | Monthly (n=6) | Non (n=4) |  |
| SM | 4.2×10^6^ (4.9×10^6^,4.3×10^6^ | | 4.1×10^6^ (4.0×10^6^,4.9×10^6^) | 4.4×10^6^ (4.2×10^6^, 4.6×10^6^) | 4.1×10^6^ (4.6×10^6^, 5.6×10^6^) | 4.0×10^6^ (4.0×10^6^,4.7×10^6^) | .841 |
| LBs | 3.8×10^6^ (3.3×10^6^,3.9×10^6^ | | 3.9×10^6^ (3.8×10^6^, 3.6×10^6^) | 4.8×10^6^ (3.5×10^6^, 4.8×10^6^) | 3.2×10^6^ (3.7×10^6^, 3.3×10^6^) | 3.5×10^6^ (3.8×10^6^,4.8×10^6^) | .597 |
| DMFT | 8(.000, 9) | | .000 (.000, 9) | .000 (.000, 5.2) | 8.5 (6, 9.5) | .000 (.000, 5.2) | .142 |
| **P <0.05* | | | | | | | |
